# Service delivery interventions to improve maternal and newborn health in low- and middle-income countries: scoping review of quality improvement, implementation research and health system strengthening approaches

**DOI:** 10.1186/s12913-023-10202-6

**Published:** 2023-11-08

**Authors:** Solange Mianda, Olamide Todowede, Helen Schneider

**Affiliations:** 1 School of Public Health & SAMRC Health Services to Systems Research Unit, University of the Western Cape, Private Bag X17, Bellville, 7535 Cape Town South Africa; 2 Institute of Mental Health, University of Nottingham, Nottingham, UK

**Keywords:** Maternal health, Newborn health, Quality improvement, Implementation science, Implementation research, Health system strengthening

## Abstract

**Introduction:**

This review explores the characteristics of service delivery-related interventions to improve maternal and newborn health (MNH) in low-and middle-income countries (LMICs) over the last two decades, comparing three common framings of these interventions, namely, quality improvement (QI), implementation science/research (IS/IR), and health system strengthening (HSS).

**Methods:**

The review followed the staged scoping review methodology proposed by Levac et al. (2010). We developed and piloted a systematic search strategy, limited to English language peer-reviewed articles published on LMICs between 2000 and March 2022. Analysis was conducted in two—quantitative and qualitative—phases. In the quantitative phase, we counted the year of publication, country(-ies) of origin, and the presence of the terms ‘quality improvement’, ‘health system strengthening’ or 'implementation science’/ ‘implementation research’ in titles, abstracts and key words. From this analysis, a subset of papers referred to as ‘archetypes’ (terms appearing in two or more of titles, abstract and key words) was analysed qualitatively, to draw out key concepts/theories and underlying mechanisms of change associated with each approach.

**Results:**

The searches from different databases resulted in a total of 3,323 hits. After removal of duplicates and screening, a total of 231 relevant articles remained for data extraction. These were distributed across the globe; more than half (*n* = 134) were published since 2017. Fifty-five (55) articles representing archetypes of the approach (30 QI, 16 IS/IR, 9 HSS) were analysed qualitatively. As anticipated, we identified distinct patterns in each approach. QI archetypes tended towards defined process interventions (most typically, plan-do-study-act cycles); IS/IR archetypes reported a wide variety of interventions, but had in common evaluation methodologies and explanatory theories; and HSS archetypes adopted systemic perspectives. Despite their distinctiveness, there was also overlap and fluidity between approaches, with papers often referencing more than one approach. Recognising the complexity of improving MNH services, there was an increased orientation towards participatory, context-specific designs in all three approaches.

**Conclusions:**

Programmes to improve MNH outcomes will benefit from a better appreciation of the distinctiveness and relatedness of different approaches to service delivery strengthening, how these have evolved and how they can be combined.

**Supplementary Information:**

The online version contains supplementary material available at 10.1186/s12913-023-10202-6.

## Introduction

Maternal and newborn mortality remains an important public health concern around the world [1, 2]. Throughout the years, improving maternal and newborn health (MNH) care has remained a global priority [3]. However, despite ongoing efforts and increased access to health services in many low-and middle-income countries (LMICs), goals of reducing maternal and newborn mortality are far from being attained [4, 5]. Additional efforts in recent years have focused not only at broadening health services coverage but also improving the quality of care provided to mothers and newborns [2, 5, 6].

An array of supply-side, health service interventions is being implemented in LMICs to improve MNH care and outcomes [7–9]. An initial scan of the literature suggests these cluster around three broad approaches to service delivery strengthening: quality improvement (QI), implementation science/research (IS/IR) and health system strengthening (HSS). The three approaches have different origins. QI applies process improvement methodologies first developed in manufacturing industry to health care [10, 11]; IS/IR emerged from the evidence-based medicine movement, and focuses on the integration of clinical guidelines into health care practice; and HSS came from the field of global health concerned with the wider health system constraints to implementation of disease-specific or programmatic interventions [12–14].

These approaches tend to use different intervention designs, concepts, terminologies, frameworks and theories. They operate within distinct professional communities, scientific journals and training streams. They also tend to engage different levels of the health system: QI is typically health facility team based (micro-level); HSS operates at meso and macro level (district, regional or national settings); IS/IR focuses on shaping the behaviour of healthcare users and/or providers while also typically referencing research methodologies such as cluster randomised trials [12, 13]. Although they have different origins and use different methodologies, the three approaches share similar goals, namely, a systematic approach to changing healthcare practice and service delivery [15]. As such, each approach, QI, IS/IR or HSS may offer ideas, concepts and methodologies that when combined could benefit the strengthening of maternal-newborn health services. However, their similarities or differences are often not appreciated or understood, and intervention design choices are seldom explicitly justified or considered in relation to alternatives [16]. Consequently, opportunities to leverage their combined strengths may be missed.

This review forms part of a multi-level service delivery initiative to improve MNH in South Africa, referred to as *Mphatlalatsane* [17]. In the inception phases of Mphatlalatsane there were debates on how to blend QI, IS/IR and HSS approaches in the design and evaluation of the project. This review was prompted by these debates and conducted to explore the scope of existing evidence on the different approaches to service delivery improvement for MNH and their methodologies and assumptions, to inform the design and/or evaluation of future complex interventions. The rationale for the review thus emerges from a practitioner perspective of decision-making in complex systems, rather than a researcher perspective of advancing knowledge on particular approaches.

## Methods

We conducted a scoping review using the methodology proposed by Levac et al. (2010) [18], to map and analyse the literature on MNH service delivery interventions. Building on previous approaches to scoping studies [19], Levac et al.’s framework emphasizes relevance to policy and practice, and the importance of aligning review purpose and research questions to review scope and strategy. This guidance resonated with our review purpose, which arose from real-world intervention design questions, and which led us to ask both objectively measurable (quantitative) and interpretive (qualitative) questions of the review. We adopted the first five stages of the Levac et al. framework (outlined below), leaving out the optional sixth stage (stakeholder consultation). A study protocol was published prior to the conduct of this review [20]. We used the preferred reporting items for systematic reviews and meta-analyses extension for scoping reviews (PRISMA-ScR) as a checklist to guide the screening and reporting [21].

### Review aim

This review explores the characteristics of service delivery-related interventions to improve MNH in LMICs over the last two decades, comparing three common framings of these interventions, namely, quality improvement (QI), implementation science/research (IS/IR), and health system strengthening (HSS).

#### Stage 1: Identifying the research questions

The overarching research question that guided this scoping review was: What are the profiles and characteristics of QI, IR and HSS or interventions used to improve MNH in LMICs?

The sub-questions were:

1. What is the distribution of approaches (QI, IR, HSS or other) in the literature on MNH service delivery interventions?
2. Who are the actors targeted for change in QI, IR or HSS interventions to improve MNH?
3. Who are the (other) health system stakeholders involved in the change processes during QI, IR or HSS interventions to improve MNH?
4. What are the services or systems areas of focus of QI, IR or HSS interventions to improve MNH?
5. What are the key constructs and concepts, frameworks and models, and theories and assumptions underlying QI, IR or HSS interventions to improve MNH?

To aid the search and selection strategy, we specified the concept, target population & health outcomes, as recommended [18] (Table 1). Table 2 provide a definition of key concepts used in the review.

**Table 1:** Concept, target group, and health outcomes

C- Concept	Quality improvement Implementation science or implementation research Health system strengthening Interventions Model or Framework Constructs and concepts Assumptions or theories
T-Target group	Actors targeted for change Stakeholders involved in change Low- and middle-income countries
Health outcomes	Maternal health Newborn health/Neonatal health (encompassing the range of processes and outcomes of service delivery-related interventions)

**Table 2:** Definition of study concepts

**Service delivery-related interventions:** refer to supply-side interventions provided through the health system and/or healthcare providers in order to improve service delivery quality and health outcomes [22].
**Quality Improvemen**t (QI): the combination efforts of multiple actors to make the changes required to leads to professional development, and better patient’s outcomes and system performance [23]. QI uses continuous methods of encouraging teams to use data to identify system gaps and apply problem solving techniques to develop timely context sensitive changes that support the effective delivery of care [24–26]. These are commonly referred to as ‘Plan-Do-Study-Act’ (PDSA) rapid cycles of change
**Implementation Science/Research (hereafter abbreviated as IR):** broadly defined as the scientific study of processes to promote the systematic uptake of promising strategies or evidence-based practice (EBPs) into routine practice and sustain them over time, to improve the quality and effectiveness of health services [12, 27, 28]. IS/IR studies outcomes, processes or factors influencing implementation [12]. Multiple theories, models and frameworks are used to implement or evaluate implementation of new scientific discoveries [29, 30]. Implementation ‘science’ and ‘research’ are used interchangeably, although implementation research has a wider meaning, associated with a range of different disciplinary traditions (e.g. policy analysis) [12, 31].
**Health Systems Strengthening (HSS)**: refers to the process of identifying and implementing changes in policy and practices in a health system, to respond better to its health and health system challenges [32]. Key elements of an HSS initiative include a system-level scope with respect to scale (cutting across multiple levels), sustainability and impacts (outcomes, equity, financial risk, and responsiveness) [33, 34].
**Low- and Middle-Income Country**: countries with gross national income per capita calculated using the World Bank Atlas method between $1,035 or less and $12,535 in 2019 (LMICs) [35, 36].
**Actors targeted for change**: refers to individuals and/or groups of people targeted by the interventions, irrespective of the approaches used [37].
**Stakeholders involved in change processes**: refer to individuals and/or groups of individuals external to the intervention or implementation setting, who promote and support the adoption of interventions, and create an enabling environment for implementation [37].

#### Stage 2: Identifying relevant studies – search strategy

This stage involved an iterative and collaborative process of searching the literature, refining the search strategy, and reviewing articles for study inclusion.

We developed a systematic search strategy using a combination of keywords and Boolean operators (AND/OR) to identify the study’s search strategy prototype. Using this strategy, we searched for English language peer reviewed articles indexed in the following electronic databases: EBSCOhost, PubMed, Web of Science, MASCOT/Wotro Map of Maternal Health Research and Google Scholar advanced search. We limited our initial search to articles published between 2000 and 2020, to capture the growth of interest in the different approaches (QI, IR and HSS) that evolved in the era of MDGs. The search was subsequently updated on 28 March 2022, to include publications in 2021 and early 2022. The search strategy was piloted to check its suitability to selected databases and keywords. A pilot sample search in PubMed is shown in Supplementary Table 1, Additional File 1. We documented each step in the search process, detailing the date, database, keywords, and the number of articles retrieved.

#### Stage 3: Selection of relevant articles

In addition to the concept, target population and health outcomes framework, inclusion and exclusion criteria guided the selection of studies (Table 3).

**Table 3:** Inclusion and exclusion criteria

Inclusion criteria	Exclusion criteria
**-** Empirical studies reporting QI, IR, HSS or other service delivery interventions to improve MNH outcomes	- Studies reporting interventions on cost-effectiveness, family planning or older children
- Studies published in English between 2000 and (28 March) 2022	- Commentaries, editorials, grey literature and reviews of interventions to improve MNH
- Studies conducted within LMICs	- Studies reporting outcomes of interventions without describing the intervention itself

We followed a two-stage process of screening: two authors (SM, OT) independently screened titles and abstracts using inclusion and exclusion criteria, and jointly resolved discrepancies; we then downloaded full texts and did another round of screening using inclusion/exclusion criteria. Over the course of charting and analysis we both added and removed articles to arrive at the final database of 231 included studies

#### Stage 4: Charting the data

A profile of the included articles was completed on an excel spreadsheet, extracting the authors, year of publication, country where the intervention was implemented, and the focus of research (maternal, newborn or both). The articles were also screened for the presence of the terms ‘quality improvement’, 'implementation science’/ ‘implementation research’ or ‘health system strengthening’ in titles, abstracts and keywords. We labelled articles as 'archetypes' when the terms QI and IR were reflected in titles, abstracts, and keywords. As there were fewer HSS papers, we included articles where HSS appeared in at least two of the three dimensions: either in the title and abstract, title and keyword or abstract and keyword. The sub-set of archetypes (n = 55) was further coded qualitatively by two authors (SM and HS) independently, documenting the nature of the intervention, including actors targeted and stakeholders involved, health systems setting, frameworks/theories adopted and key elements of interventions.

The data charting and qualitative coding (completed data sheets) is available on request from the authors.

#### Stage 5: Collating, summarizing, and reporting of results

Data analysis was conducted in two steps: 1) The full database of 231 articles formed the basis of a descriptive quantitative analysis of publication dates, country(ies) of study and profile and overlap of approaches, conducted in SPSS. 2) The qualitative coding sheets compiled by SM and HS were combined, and through dialogue between the two authors, key characteristics of and patterns in each set of archetypes identified. These were then compared and contrasted with the other archetypes, and implications for policy and practice formulated.

## Results

**Fig. 1:**
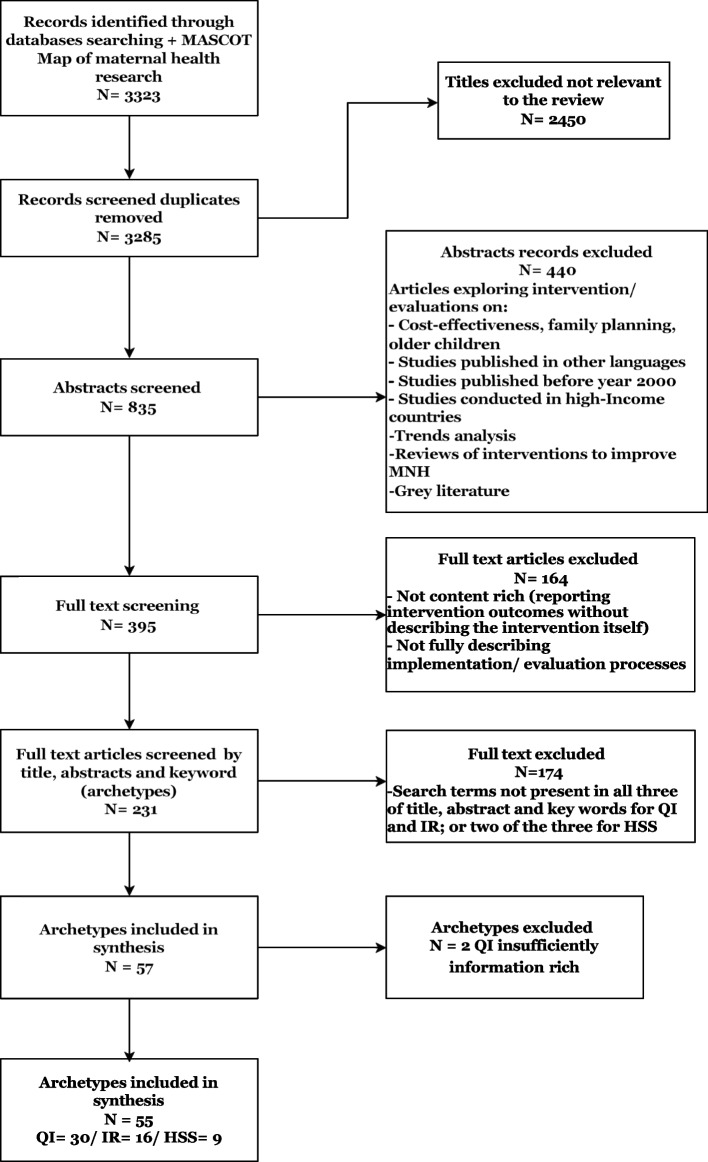
Flow diagramme of studies included and excluded

The searches from different databases resulted in a total of 3,323 hits (EBSCOhost = 137, PubMed = 1,475, Web of Science = 1,650, MASCOT/Wotro Map of Maternal Health Research = 37; and citation search = 24). After removal of duplicates and the first two stages of screening, a total of 231 potentially relevant articles remained for data extraction (See flow chart in Fig. 1). The third stage of screening for approaches (QI, IR, HSS) in the title, abstract and keywords led to 57 articles we identified as archetypes for qualitative analysis (Fig. 1).

### Distribution of studies reviewed

**Fig. 2:**
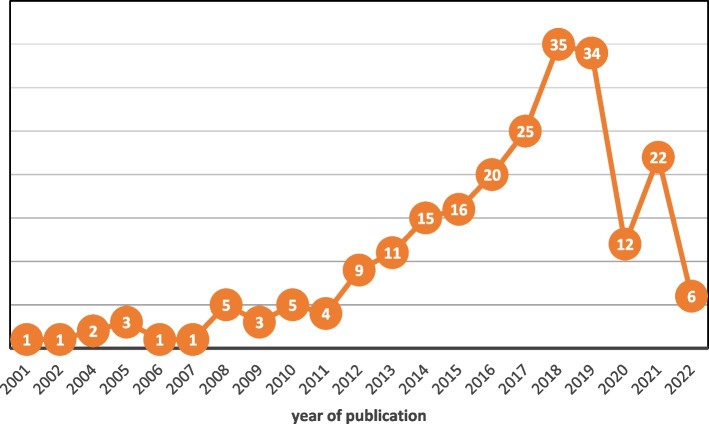
Number of publications by year (*n* = 231)

The distribution of articles by year, country and focus are reported below. From 2012 onwards, there was a steady growth in the number of published articles, plateauing in 2018 (*n* = 35) and 2019 (*n* = 34) and dropping to lower levels in 2020/1 (Fig. 2). More than half (*n* = 134, 58%) were published from 2017 onwards.

**Fig. 3:**
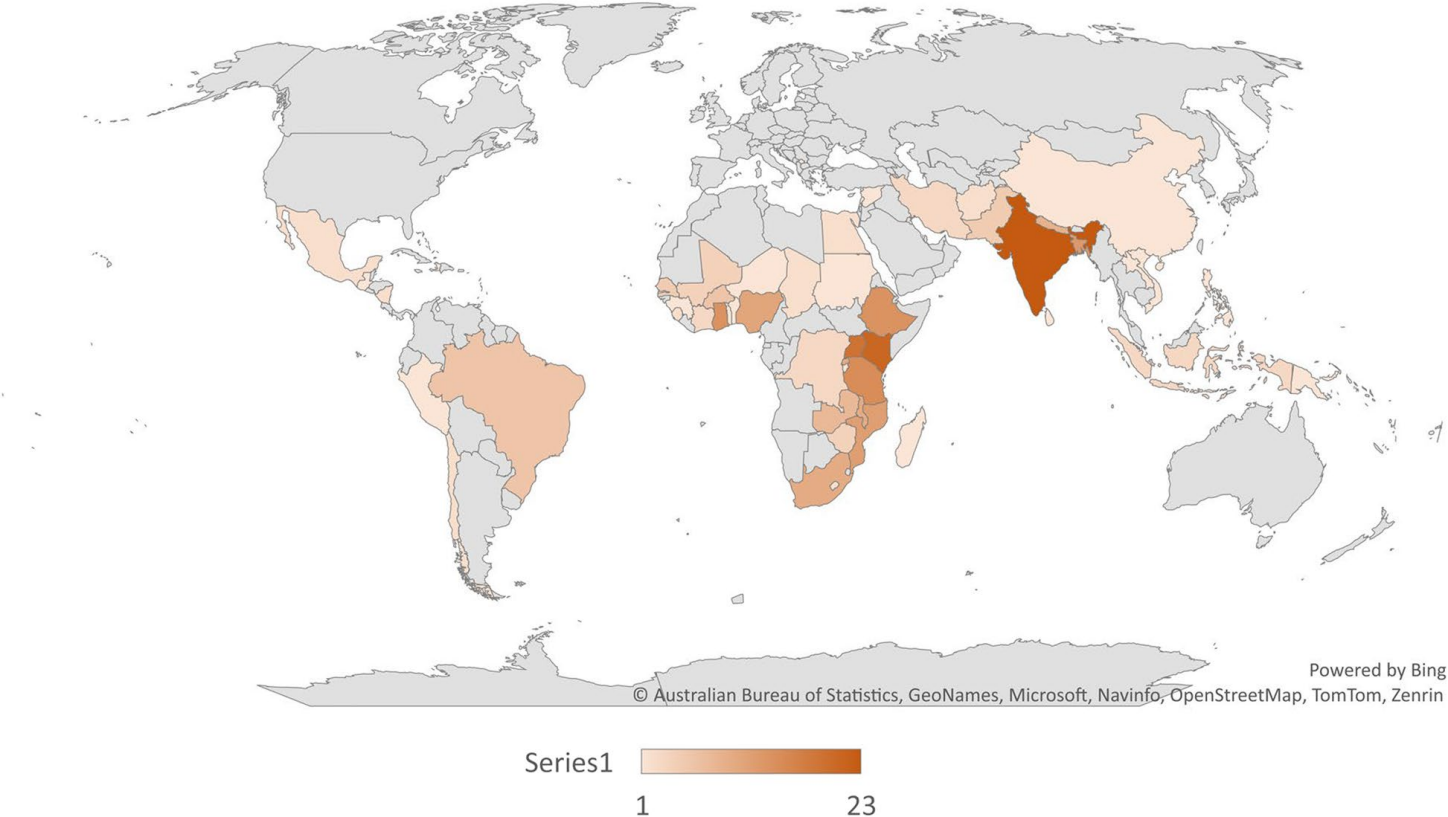
Countries where interventions were implemented

The articles reported on interventions in LMICs across the globe, with India (*n* = 23) and Kenya (*n* = 21) the most frequently represented (Fig. 3).

**Fig. 4:**
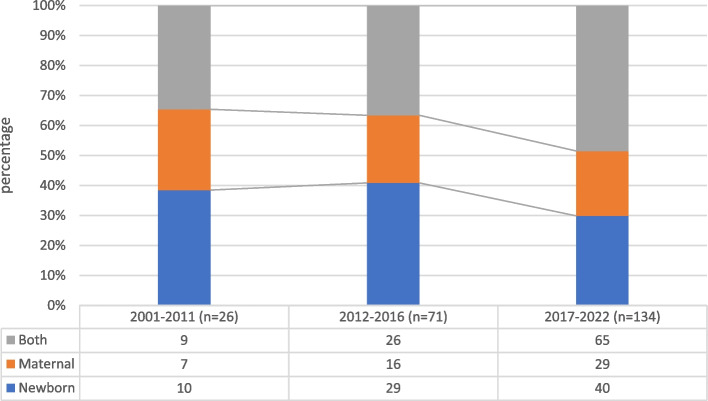
Distribution of field of research over time

The focus of interventions (maternal, newborn or both) shifted over the years: while there was an overall increase in studies in all three categories, the ratio between them changed, from a singular to a combined focus (Fig. 4).

### Profile of interventions to strengthen maternal and newborn health services in LMIC papers

Presence of terms in one or more of the title, abstract and keywords showed a preponderance of IR (147 studies with at least one mention), not surprisingly as IS/IR is a generic category encompassing a wide range of approaches [12], followed by QI (86 mentions), and HSS (29 mentions). However, QI approaches were most likely to be referenced in all three elements of the paper (*n* = 32) compared to 20 for IR/IS and only two for HSS (Supplementary Fig. 1). Twelve percent of papers made no reference to any of the three approaches, using more general descriptors of intervention e.g. strategy, programme or initiative (Supplementary Fig. 2). These studies were picked up by search terms such as ‘health system’ and ‘health system intervention’, but not ultimately classified as HSS or one of the other two approaches.

Studies often referenced more than one approach, most commonly a combination of QI and IR (*n* = 43). Five studies referenced all three approaches at least once and four papers were classified as archetypes of two approaches.

### Qualitative analysis of archetypes

Of the 57 articles identified as archetypes, 55 were analysed qualitatively. Two QI archetypes were excluded as insufficiently information rich. The remaining 55 included 30 articles on QI, 16 articles on IR and 9 articles on HSS approaches, respectively. Four IR archetypes were also archetypes of, and assigned to, the other two categories. The 55 papers were published in 30 general and subject specific journals, with Implementation Science (*n* = 6), BMC Health Services Research (*n* = 5) and BMC Reproductive Health (*n* = 5) being the most common (Supplementary Fig. 3). The papers were a mix of intervention descriptions, process and effectiveness/outcome evaluations (or protocols for these), retrospective case studies of implementation factors and analyses of intervention scale up. They encompassed both qualitative and quantitative methodologies, with a strong influence of (quasi-)experimental designs such as cluster hybrid implementation-effectiveness studies. While the interventions described were all ultimately concerned with shaping frontline health care provider practice, they addressed a range of micro, meso and macro level factors and players, depending on the purpose of the study (e.g. effectiveness, scale up, sustainability) and/or assumptions of change.

Detailed analysis of the MNH service delivery interventions in archetypes of the three approaches revealed distinct patterns but also considerable variation within and overlap between approaches. Table 4 compares the three approaches, including models/frameworks, interventions, key ideas and overall orientations discerned in the studies. QI emerged as the dominant approach from 2019 onwards (see also Supplementary Fig. 4). Studies were distributed across four continents, and most often reported on a single country, in contrast to the HSS studies which were in Africa only, and which included three large multi-country initiatives.

**Table 4:** Comparisons of the three approaches

Approaches	Quality Improvement	Implementation Research	Health System Strengthening
Number of articles	30	16	9
Publications since 2019	17	7	4
Field of intervention	Newborn: 11 Maternal: 10 Both: 9	Newborn: 4 Maternal: 6 Both: 6	Newborn: 3 Maternal: 0 Both: 6
Continental distribution	Africa, Asia, South America 3/30 studies in two countries	Africa, Asia, South America 3/16 multi-country (2 × 3 countries, 1 × 5 countries)	Africa only 3/9 multi-country (2 × 4 countries, 1 × 5 countries)
Models and Frameworks	IHI Collaborative QI model POCQI: WHO Point of Care QI Model SBM-R: Standards Based Management and Recognition EQUIP: enhanced quality management using information power MESH-QI: mentorship, enhanced supervision for health care + QI	Stages of change COM-B: Capability, Opportunities, Motivation Behaviour CFIR: Consolidated Framework for Implementation Research PARIHS: Promoting Action on Research Implementation in Health Services TDF: Theoretical Domains Framework RE-AIM: Research, effectiveness, adoption, implementation and maintenance	Tanzania Essential Health Information Project (TEHIP) Health System Building Blocks Step-wise, multi-stakeholder Whole of health system
Key features of interventions	Plan-Do-Study-Act (PDSA) • Participatory • Multi-disciplinary teams (MDT) • Local problem-solving • Problem analysis tools • Change ideas • Rapid cycle tests of change • Targets • Monitoring & data use Measurement based • Standards • Targets • Audit and feedback • Household and facility surveys Meso-level support • Training (low density, high frequency), on-site, simulations • Supportive supervision, mentoring, coaching • Resourcing, incl. performance-based financing, supplies • Real-time data, report cards, registers Macro-level scale up • Cross-site learning collaboratives • Planning	Community based interventions • Recruitment and training of volunteers, CHWs • Home-based services • mHealth • Community participation (dialogues, leadership buy-in) • Traditional birth attendants • Community financing and governance Facility level strategies • e-health tracking • Group antenatal care • Clinical care bundles • Birth companions • Training, guidelines, simulation • Equipment, supplies, infrastructure • Team work, champions • Steering committees Meso-level interventions • Manager training & support • Resourcing • Referral • Supportive supervision, on site- mentoring Macro-level scale up • Senior leadership support, champions • Centres of excellence • Training cascades • Free maternal care Participatory intervention design • Interviews, surveys, workshops	Macro-level strategies • Senior leadership commitment and champions • Resource mobilisation, financing, removal of user fees • Specialist outreach, referral systems • Evidence based care packages Meso-level strategies • District level evidence-based planning • Resource mobilisation, including financing • Expanded services • Health information systems • Supply chains • Leadership, district management development • HR recruitment and retention, task shifting Community based strategies • Community based care, nurses and CHWs • Information & feedback • Local political leaders Facility based strategies • Infrastructure upgrading • Training • Performance review • Performance based financing • QI Teams and processes • Telehealth • Clinical mentorship, coaching Participatory designs • Teams analyse data and prioritise interventions • ToC, logic models • Mapping patient pathways • Co-production
Key ideas/ constructs	Collaboration, team work, action learning (PDSA), local problem solving; data use; standards and targets	Uptake of evidence-based interventions Evidence-practice gap, behaviour change Theory-based, effectiveness-implementation evaluation	Whole system perspectives Demand and supply Multi-level action
Overall orientation	Defined processes focused on provider problem solving with meso-level support, macro-level scale up	Defined interventions and/or processes for implementation of evidence-based practice, engaging both providers and users; theory-based evaluation	Enabling systems, multi-level, whole system perspectives, health system inputs (HR, financing, supply chains), resourcing and incentives

### Models and interventions used in included studies

#### Quality improvement models

The QI studies adopted a mix of models. Many drew on the Institute for Healthcare Improvement (IHI) framework [38, 39], also referred to as QI collaboratives [40], or the Breakthrough Series Model [38]. The IHI model is centred on iterative ‘Plan-Do-Study-Act (PDSA)’ cycles, implemented by frontline health care teams, and has evolved into a multifaceted quality improvement methodology, including diagnostic and monitoring processes and tools, and scale up processes [41]. Drawing on the same core ideas of facility team-based problem identification and PDSA cycles, the WHO Point of Care QI (POCQI) model was the basis of several studies, particularly in India. It was developed by the WHO South East Asian Regional Office (SEARO) specifically for MNH care [42–44]. One paper referenced Continuous Quality Improvement (CQI), following a similar approach [45]. A number of QI studies adopted processes of standard setting and regular measurement, such as the Standards Based Management and Recognition (SBM-R) model [26, 46, 47], addressing specific bottlenecks arising from the measurement process. The EQUIP (Enhanced Quality Management using Information Power) study combined standards, measurement and PDSA processes [48]. Similarly, the MESH-QI (Management, Enhanced Supervision for Health Care and QI) combined PDSA cycles with meso-level supportive strategies and training [49]. QI studies drew on explanatory frameworks from the field of IR (see below) such as Promoting Action on Research Implementation in Health Services (PARIHS) and the Consolidated Framework for Implementation Research (CFIR) [39, 50, 51].

The QI studies addressed both specific issues (e.g. antibiotic usage, thermoregulation in a neonatal ICU, postpartum haemorrhage) and more general problems (e.g. reducing perinatal mortality). The interventions were for the most part focused on micro, facility level teams and participatory processes (PDSA cycles, problem analysis, change ideas, monitoring etc.) [52]. Some also considered meso-level strategies for supporting facility teams [49], while others included participatory processes (‘learning collaboratives’) at district and other levels to scale up QI interventions [53–55]. In sum, the overall orientation of the QI interventions was on defined processes aimed at health facility teams with supportive and scale up processes at meso and macro levels.

#### Implementation research models

In contrast to the QI studies, the IR study interventions were more diverse, with a range of entry points (community and facility based, meso and macro-level, sometimes in combination), and adopting a variety of tools (e.g. e-health, checklists) and mechanisms (e.g. CHWs, financial incentives) (Table 4). Similar to QI studies their interventions included both specific (e.g. birth companions, kangaroo mother care) and multi-faceted packages (combining community and facility-based interventions) [56]. Several were concerned with explaining implementation at scale [57–59]. Frameworks guiding change in these studies included the three-level Stages-of-Change model [60]: pre-implementation (readiness of stakeholders), implementation (readiness of system) and institutionalization; and the ‘The Capability, Opportunity, Motivation, Behaviour (COM-B)’ model to implement clinical care bundles for post-partum haemorrhage. COM-B stands for Capability (training, on-site simulations), Opportunities (physical infrastructure and resources), and Motivation (champions, actionable intelligence) for Behaviour [61]. Recognising the importance of adapting interventions to local contexts, the latter included an extensive phase of formative research and co-design of interventions, followed by adaptive cycles of implementation, not unlike a PDSA cycle.

In the main, theories reported in the IR studies were descriptive or explanatory models of change rather than prospective guides to intervention implementation (in contrast to the QI process models). Frameworks included the descriptive account of implementation, such as the RE-AIM (Reach, Effectiveness, Adoption, Implementation and Maintenance) heuristic [62], or constructs such implementation fidelity, acceptability and intervention strength [61–64]. Several studies sought to explain (non)implementation using frameworks such as the Consolidated Framework Implementation Research (CFIR) [65], The Promotion Action of Research in Health (PARIHS) [58], and Theoretical Domains Framework (TDF) [60]. These frameworks provide a structured means for assessing the barriers and facilitators of implementation, and to generate context-specific recommendations for further implementation of evidence-based interventions.

IR studies thus approached the mechanisms and drivers of behaviour and system change with an array of implicit assumptions, with the common goal of testing different ways of achieving evidence-based practice through rigorous and theory-based evaluation designs.

#### Health systems strengthening models

The HSS Interventions focused on systems-level enablers, implicitly or explicitly drawing on the WHO Health System Building Blocks framework [66], altering system level inputs such as financing, human resource, leadership and governance, infrastructure, supply chain mechanisms and information, sometimes in combination with specific facility-based QI strategies [67]. The Ghana Essential Health Intervention Packages (GEHIP) was a district and regional planning, resource allocation and leadership methodology supporting the implementation of community-based service delivery referred to as CHPS [68]. The rationale for GEHIP was that “the CHPS initiative was originally conceived as a community-based trial focused on identifying the best way of delivering services and sustaining community engagement for primary health care rather than a systems initiative”. Similar to developments in IR, recent approaches to HSS include participatory designs, in which "activities engage stakeholders and build relationships to ensure coproduction and ownership of HSSIs [health system strengthening interventions]" [69]. For example, Seward et al [69] outline a structured participatory process that involves mapping a patient’s journey through the health system and the associated health system bottlenecks, followed by a joint ‘Theory of Change’ workshop providing contextualised recommendations. Similarly, Kung'u et al [36] adopt a staged approach to enlisting support and participation in four African countries, analysing local contexts and jointly developing designs.

Overall, the core orientation of HSS is whole of health system, multi-level interventions to create enabling environments for change at the micro-level. HSS focuses on health system inputs and multi-stakeholder collaborative processes, engaging both a ‘demand’ (user) and ‘supply’ (system) sides of the system.

Across all three approaches (in particular the HSS studies), there was a large footprint of external donors, global health institutions, northern universities or international NGOs in intervention design and implementation.

## Discussion

The review started from the premise that there are distinct schools of thought characterised as QI, IR/IS and HSS—in strengthening MNH services in LMICs, and the value of understanding the approaches in design choices. We sought to characterise these approaches and their respective assumptions of change in the published literature that self-identified with each of these approaches, as reflected in titles, abstracts and key words.

Our review discerned broad patterns associated with each approach, but also considerable fluidity and overlap between them. QI had the clearest identity and offered the most direct approach to managing change with a key focus on micro level teams. Two specific orientations were evident in the QI approach: the PDSA cycle as a structured methodology for activating facility teams and processes, and a 'standards and measurement-based’ approach. This reflects the broader debates in quality field, between assurance (measurement) and improvement (process) approaches [70].

There is growing recognition that micro-level initiatives by themselves are not sufficient and need to be combined with quality strategies and planning at multiple levels of the health system [71, 72]. As pointed out by Dixon-Woods & Martin [73], "too little has been spent on the organisational strengthening needed to make improvement.” This realisation was evident in the studies where QI interventions involved actors at meso and macro levels for support and resource mobilisation.

QI initiatives are increasingly used to improve MNH in LMICs [74–77]. While they appear technically easy and appropriate in resource constrained areas, interventions do not always embed or sustain within health systems, [77–79] and are constrained by a lack of political will and inadequate buy-in from leaders and resources to address problems, and the skills and time to apply QI methodologies [80, 81]. To sustain the gains from QI initiatives, greater learning from and sharing of QI implementation within and across levels, organizations, countries and regions as well as institutionalisation of quality is required [75].

The other two approaches were associated with a wide variety of interventions and pathways of change. IS/IR is a broad category [12], and even in the narrower sub-field of IS, the reported interventions solved a range of implementation problems in diverse contexts [12, 13]. Studies drew on an extensive repertoire of theory and frameworks [12–14], although these were applied less as theories of change than explanatory theories of factors that affected implementation outcomes [82]. The IR studies thus offered explanatory models and rigorous approaches to evaluation (effectiveness-implementation studies), and a large menu of possible interventions, but limited guidance on possible pathways or mechanisms of change [12, 31, 83, 84]. This may account for the growing popularity of QI methodologies that provide such a structured change methodology.

The HSS models recognised the need for ‘above facility’ change processes in the meso and macro levels of health systems, and tended to be holistic, embodying some degree of complexity and co-production, and were intrinsically multi-level in orientation. Applying a HSS lens on service delivery strengthening requires an understanding of the dynamic interactions between the building blocks of the health system, organisations and actors within specific contexts [32]. The multilevel orientation (actors, stakeholders, health settings) [33, 34] of HSS can strengthen the system, and foster better performance through supportive leadership, engaged teams, well supplied facilities, trained healthcare providers and supportive supervision systems [67]. However, they are less oriented to micro-level change processes.

All approaches converged on the need for multi-level, multi-component interventions, and increasingly, on locally developed, participatory and context specific designs. These are appropriate to change in complex health systems [85–87], evolving from linear chains of cause-and-effect, towards engaging interconnected elements holistically [88–91].

Returning to the practical problem of intervention design posed at the start of this paper, a synthesis of insights from the different approaches would recognise that there are no magic bullets [72] or single answers to strengthening MNH services in LMICs. Programmes ideally draw from different approaches and ‘logics’ in flexible ways [92], and are tailored to specific contexts. For example, a combination of facility-level PDSA cycles, participatory analyses of barriers to implementation, jointly developed theories of change, planning and resource allocation at meso level, and macro-level leadership and policy, might provide the best overall approach to strengthening MNH services. Interventions ultimately need to find resonance within local health systems and respond to a felt need to be assimilated and adopted.

### Limitations

Many of the studies in this review, with important exceptions, emerged from global health institutions with an orientation towards testing the effectiveness of interventions, rather than their sustainable implementation at scale. The peer reviewed literature generally does not report on the experiential and practice knowledge of decision-makers and implementers, potentially offering different understandings of, and approaches, to MNH service delivery improvement [93]. This points to a gap in scholarship, as well as the limits of a review such as this.

We acknowledge that our search strategy and screening process, starting with an a priori hypotheses on three approaches, may have missed relevant service delivery interventions. However, it is likely that generic terms such as ‘service’ or ‘system’ would have led us to key approaches. Our screening also focused on supply side interventions focused on health care providers. While these intervention packages at times also included community-based strategies, purely demand side approaches (most notably participatory women’s groups) were beyond the scope of this review. However, these approaches have been influential globally [94], can be a trigger for quality improvement [95], and need to be considered in intervention programmes. Finally, although the review identified studies across continents, the emphasis on English-language papers may have introduced a language bias, limiting diverse perspectives. The inclusion of grey literature, even if hard to source and manage, could have allowed for a greater range of insights on service delivery improvement.

## Conclusion

This review examined and compared three widely used approaches to strengthen MNH service delivery: QI, IS/IR and HSS in LMICs. As hypothesised, we were able to identify distinct targets, theories and assumptions in the three approaches while also acknowledging considerable fluidity between approaches. Programmes to improve MNH outcomes will benefit from a better appreciation of the distinctiveness and relatedness of different approaches to service delivery strengthening, how these have evolved and how they can be combined. Further exploration is needed into the practicality and effectiveness of combining these approaches in multi-level interventions.

### Supplementary Information

**Additional file 1:** **Supplementary Table 1.** Search String strategy [Pilot sample in PubMed]. **Supplementary Figure 1.** Terms appearing in title or keywords or abstracts (*n*=231). **Supplementary Figure 2.** Percent of publications where terms appear at least once (*n*=231). **Supplementary Figure 3.** Distribution of journals publishing archetypes, showing archetype distribution (*n*=55) (blue = QI; orange = IR; grey = HSS). **Supplementary Figure 4.** Distribution of archetypes over time (*n*=55).

## Data Availability

All data generated or analyzed during this study are included in this published article.

## Abbreviations

CHWs: Community health workers
EBP: Evidence-based-practices
HSS: Health system strengthening
IS: Implementation science
IR: Implementation Research
LMICs: Low- and Middle-Income Countries
MNH: Maternal and newborn health
NGO: Non-profit organization
PDSA: Plan-do-study-act
QI: Quality improvement
SPSS: Statistical package for the social sciences
