# Supplementary material for: Service delivery interventions to improve maternal and newborn health in low- and middle-income countries: scoping review of quality improvement, implementation research and health system strengthening approaches
Source: BMC Health Serv Res. 2023 Nov 8;23:1223. doi: 10.1186/s12913-023-10202-6 (PMC10634015; doi:10.1186/s12913-023-10202-6)
Supplement: Supplementary file 1 — Additional file 1: Supplementary Table 1. Search String strategy [Pilot sample in PubMed]. Supplementary Figure 1. Terms appearing in title or keywords or abstracts (n=231). Supplementary Figure 2. Percent of publications where terms appear at least once (n=231). Supplementary Figure 3. Distribution of journals publishing archetypes, showing archetype distribution (n=55) (blue = QI; orange = IR; grey = HSS). Supplementary Figure 4. Distribution of archetypes over time (n=55). [file 12913_2023_10202_MOESM1_ESM.docx]

**Supplementary Table 1. Search String strategy [Pilot sample in PubMed]**

| *Pubmed* | |
| --- | --- |
| 1 | developing countr*[MH] OR developing countr*[TIAB] OR developing nation*[TIAB] or developing world[TIAB] |
| 2 | least developed countr*[TIAB] OR least developed nation*[TIAB] OR least developed world[TIAB] OR least-developed countr*[TIAB] OR least-developed nation*[TIAB] OR less-developed countr* OR less-developed nation*[TIAB] OR less developed countr*[TIAB] OR less developed nation*[TIAB] |
| 3 | under-developed countr*[TIAB] OR under developed countr*[TIAB] OR underdeveloped countr*[TIAB] OR under-developed nation*[TIAB] OR under developed nation*[TIAB] OR underdeveloped nation*[TIAB] OR under-developed world[TIAB] OR under developed world[TIAB] OR underdeveloped world[TIAB] OR under-developed econom*[TIAB] OR under developed econom*[TIAB] OR underdeveloped econom*[TIAB] |
| 4 | third world countr*[TIAB] OR third world nation*[TIAB] OR third-world countr*[TIAB] OR third-world nation*[TIAB] |
| 5 | low- and middle-income countr*[TIAB] OR low and middle income countr*[TIAB] OR low- and middle-income nation*[TIAB] OR low and middle income nation*[TIAB] OR low- and middle-income world[TIAB] OR low and middle income world[TIAB] OR low- and middle-income econom*[TIAB] OR low and middle income econom*[TIAB] OR low income countr*[TIAB] OR middle income countr*[TIAB] OR low-income countr*[TIAB] OR middle-income countr*[TIAB]OR low income nation*[TIAB] OR middle income nation*[TIAB] OR low-income nation*[TIAB] OR middle-income nation*[TIAB] OR low income world[TIAB] OR middle income world[TIAB] OR low-income world[TIAB] OR middle-income world[TIAB] OR low income econom*[TIAB] OR middle income econom*[TIAB] OR low-income econom*[TIAB] OR middle-income econom*[TIAB] |
| 6 | LIC[TIAB] OR LICs [TIAB] OR MIC[TIAB] OR MICs [TIAB] OR LMIC[TIAB] OR LMICs[TIAB] OR LAMIC[TIAB] OR LAMICs[TIAB] OR LAMI countr*[TIAB] |
| 7 | Transitional countr*[TIAB] OR Transitional econom*[TIAB] OR Transition countr*[TIAB] OR Transition econom*[TIAB] |
| 8 | Asia[MH] OR Africa[MH] OR South America[MH] OR Caribbean region[MH] OR Central America[MH] |
| 9 | Afghanistan[TIAB] OR Albania[TIAB] OR Algeria[TIAB] OR American Samoa[TIAB] OR Angola[TIAB] OR Antigua[TIAB] OR Barbuda[TIAB] OR Argentina[TIAB] OR Armenia[TIAB] OR Azerbaijan[TIAB] OR Bangladesh[TIAB] OR Belarus[TIAB] OR Byelarus[TIAB] OR Byelorussia[TIAB] OR Belorussia[TIAB] OR Belize[TIAB] OR Benin[TIAB] OR Bhutan[TIAB] OR Bolivia[TIAB] OR Bosnia[TIAB] OR Herzegovina[TIAB] OR Hercegovina[TIAB] OR Bosnia-Herzegovina[TIAB] OR Bosnia-Hercegovina[TIAB] OR Botswana[TIAB] OR Brazil[TIAB] OR Brasil[TIAB] OR Bulgaria[TIAB] OR Burkina[TIAB] OR Upper Volta[TIAB] OR Burundi[TIAB] OR Urundi[TIAB] OR Cambodia[TIAB] OR Republic of Kampuchea[TIAB] OR Cameroon[TIAB] OR Cameroons[TIAB] OR Cape Verde[TIAB] OR Central African Republic[TIAB] OR Chad[TIAB] OR Chile[TIAB] OR China[TIAB] OR Colombia[TIAB] OR Comoros[TIAB] OR Comoro Islands[TIAB] OR Comores[TIAB] OR Congo[TIAB] OR DRC[TIAB] OR Zaire[TIAB] OR Costa Rica[TIAB] OR Cote d'Ivoire[TIAB] OR Ivory Coast[TIAB] OR Cuba[TIAB] OR Djibouti[TIAB] OR Obock[TIAB] OR French Somaliland[TIAB] OR Dominica[TIAB] OR Dominican Republic[TIAB] OR Ecuador[TIAB] OR Egypt[TIAB] OR United Arab Republic[TIAB] OR El Salvador[TIAB] OR Eritrea[TIAB] OR Ethiopia[TIAB] OR Fiji[TIAB] OR Gabon[TIAB] OR Gabonese Republic[TIAB] OR Gambia[TIAB] OR Georgia[TIAB] OR Ghana[TIAB] OR Gold Coast[TIAB] OR Grenada[TIAB] OR Guatemala[TIAB] OR Guinea[TIAB] OR Guinea-Bissau[TIAB] OR Guiana[TIAB] OR Guyana[TIAB] OR Haiti[TIAB] OR Honduras[TIAB] OR India[TIAB] OR Indonesia[TIAB] OR Iran[TIAB] OR Iraq[TIAB] OR Jamaica[TIAB] OR Jordan[TIAB] OR Kazakhstan[TIAB] OR Kenya[TIAB] OR Kiribati[TIAB] OR Republic of Korea[TIAB] OR North Korea[TIAB] OR DPRK[TIAB] OR Kosovo[TIAB] OR Kyrgyzstan[TIAB] OR Kirghizstan[TIAB] OR Kirgizstan[TIAB] OR Kirghizia[TIAB] OR Kirgizia[TIAB] OR Kyrgyz[TIAB] OR Kirghiz[TIAB] OR Kyrgyz Republic[TIAB] OR Lao[TIAB] OR Laos[TIAB] OR Latvia[TIAB] OR Lebanon[TIAB] OR Lesotho[TIAB] OR Basutoland[TIAB] OR Liberia[TIAB] OR Libya[TIAB] OR Lithuania[TIAB] OR Macedonia[TIAB] OR Madagascar[TIAB] OR Malagasy Republic[TIAB] OR Malawi[TIAB] OR Nyasaland[TIAB] OR Malaysia[TIAB] OR Malaya[TIAB] OR Malay[TIAB] OR Maldives[TIAB] OR Mali[TIAB] OR Marshall Islands[TIAB] OR Mauritania[TIAB] OR Mauritius[TIAB] OR Mayotte[TIAB] OR Mexico[TIAB] OR Micronesia[TIAB] OR Moldova[TIAB] OR Moldovia[TIAB] OR Mongolia[TIAB] OR Montenegro[TIAB] OR Morocco[TIAB] OR Mozambique[TIAB] OR Myanmar[TIAB] OR Burma[TIAB] OR Namibia[TIAB] OR Nepal[TIAB] OR Nicaragua[TIAB] OR Niger[TIAB] OR Nigeria[TIAB] OR Pakistan[TIAB] OR Palau[TIAB] OR Palestine[TIAB] OR Panama[TIAB] OR Papua New Guinea[TIAB] OR Paraguay[TIAB] OR Peru[TIAB] OR Philippines[TIAB] OR Romania[TIAB] OR Rumania[TIAB] OR Roumania[TIAB] OR Russia[TIAB] OR Russian Federation[TIAB] OR USSR[TIAB] OR Soviet Union[TIAB] OR Union of Soviet Socialist Republics[TIAB] OR Rwanda[TIAB] OR Ruanda-Urundi[TIAB] OR Samoa[TIAB] OR Samoan Islands[TIAB] OR Sao Tome[TIAB] OR Principe[TIAB] OR Senegal[TIAB] OR Serbia[TIAB] OR Montenegro[TIAB] OR Yugoslavia[TIAB] OR Seychelles[TIAB] OR Sierra Leone[TIAB] OR Solomon Islands[TIAB] OR Somalia[TIAB] OR South Africa[TIAB] OR Sri Lanka[TIAB] OR Ceylon[TIAB] OR Saint Kitts[TIAB] OR St Kitts[TIAB] OR Saint Christopher Island[TIAB] OR Nevis[TIAB] OR Saint Lucia[TIAB] OR St Lucia[TIAB] OR Saint Vincent[TIAB] OR St Vincent[TIAB] OR Grenadines[TIAB] OR Sudan[TIAB] OR Suriname[TIAB] OR Surinam[TIAB] OR Swaziland[TIAB] OR Syria[TIAB] OR Syrian Arab Republic[TIAB] OR Tajikistan[TIAB] OR Tadzhikistan[TIAB] OR Tadjikistan[TIAB] OR Tanzania[TIAB] OR Thailand[TIAB] OR Timor-Leste[TIAB] OR East Timor[TIAB] OR Togo[TIAB] OR Togolese Republic[TIAB] OR Tonga[TIAB] OR Tunisia[TIAB] OR Turkey[TIAB] OR Turkmenistan[TIAB] OR Turkmenia[TIAB] OR Tuvalu[TIAB] OR Uganda[TIAB] OR Ukraine[TIAB] OR Uruguay[TIAB] OR Uzbekistan[TIAB] OR Vanuatu[TIAB] OR New Hebrides[TIAB] OR Venezuela[TIAB] OR Vietnam[TIAB] OR Viet Nam[TIAB] OR West Bank[TIAB] OR Gaza[TIAB] OR Yemen[TIAB] OR Zambia[TIAB] OR Zimbabwe[TIAB] OR Rhodesia[TIAB] |
| 10 | 1 OR 2 OR 3 OR 4 OR 5 OR 6 OR 7 OR 8 OR 9 |
| 11 | quality improvement [MeSH Terms] OR quality improvement [TIAB] OR quality of Health care Improvement [TIAB] OR improve quality [TIAB] OR "quality"[TIAB] AND improvement [TIAB] |
| 12 | Health system strengthening [MeSH Terms] OR Health system*[TIAB] OR Health system strengthen* [TIAB] OR HSS[TIAB] OR Health system intervention*[TIAB] |
| 13 | Implementation science [MeSH Terms] OR implementation research [MeSH Terms] OR implementation science [TIAB] OR Implementation research [TIAB] |
| 14 | 11 OR 12 OR 13 |
| 15 | Maternal Health [MeSH Terms] OR Maternal Health Service*[MeSH Terms] OR Maternal Health and Child Health [MeSH] OR Maternal Child Health [MeSH] OR Maternal Health [TIAB] OR Maternal Health Service*[TIAB] OR Maternal health and child health [TIAB] OR Maternal Child Health [TIAB] |
| 16 | Child Health [MeSH Terms] OR Child Health Service*[MeSH Terms] OR Child Health [TIAB] OR Child Health Service*[TIAB] |
| 17 | Newborn [MeSH Terms] OR Infant*[MeSH Terms] OR Perinatal [MeSH Terms] OR Infant Health service*[MeSH Terms] OR Infant Health [TIAB] OR Perinatal Health [TIAB] OR Newborn Health Service*[TIAB] OR Baby health [TIAB] OR Neonatal health [TIAB] Perinatal Health service [TIAB] |
| 18 | 15 OR 16 OR 17 |
| 19 | Theory [MeSH Terms] OR Social Theory [MeSH Terms] OR Framework [MeSH Terms] OR concept[MeSH Terms] OR Theoretical framework[MeSH Terms] OR conceptualization*[MeSH Terms] OR Model*[MeSH Terms] OR Theory[TIAB] OR Social Theory [TIAB] OR Framework [TIAB] OR concept[TIAB] OR Theoretical framework[TIAB] OR conceptualization*[TIAB] OR Model*[TIAB] |
| 20 | 10 AND 14 AND 18 AND 19 |
| 21 | Limit 20 to English language, publication type=case reports, comparative study, evaluation studies, journal article, meta-analysis, review, systematic reviews, 2000 – till date |

|  |  |
| --- | --- |

**Supplementary Figure 1: Terms appearing in title or keywords or abstracts (n=231)**

**Supplementary Figure 2: Percent of publications where terms appear at least once (n=231)**

* studies picked up by search terms ‘health system’ and ‘health system intervention’, but not ultimately classified as HSS


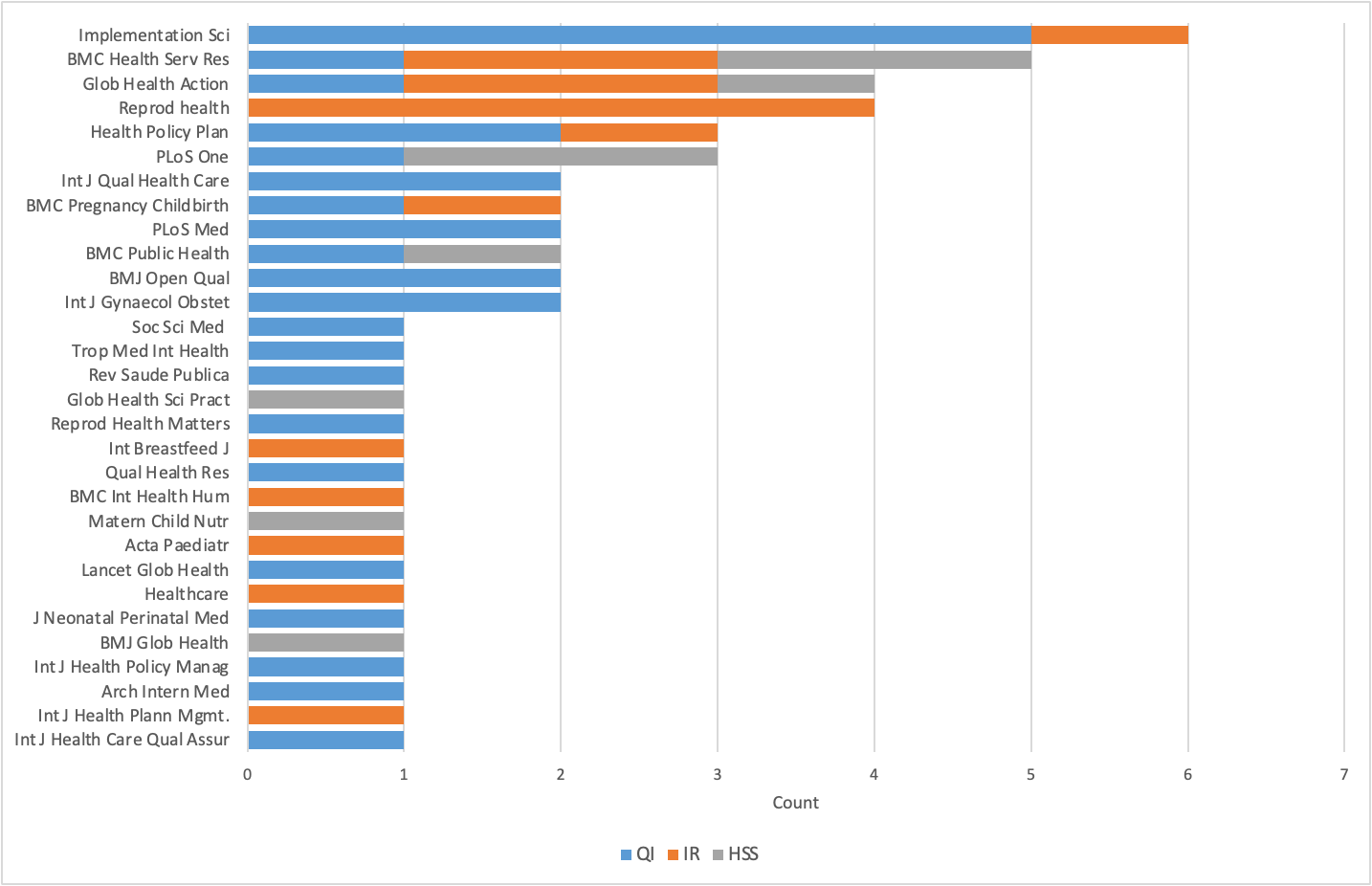


**Supplementary Figure 3: Distribution of journals publishing archetypes, showing archetype distribution (n=55) (blue = QI; orange = IR; grey = HSS)**

**Supplementary Figure 4: Distribution of archetypes over time (n=55)**
